# Breaking the Y

**DOI:** 10.1371/journal.pgen.1008109

**Published:** 2019-05-23

**Authors:** Guillaume Holzer, Wolfram Antonin

**Affiliations:** Institute of Biochemistry and Molecular Cell Biology, Medical School, RWTH Aachen University, Aachen, Germany; The University of North Carolina at Chapel Hill, UNITED STATES

Nuclear pore complexes (NPCs) are large protein assemblies in the nuclear envelope acting as portals for protein and RNA transport between the nucleus and cytoplasm. Because of their universal function in all nucleated cells, the overall structure of NPCs is thought to be evolutionarily largely conserved [1, 2]. Its core structure is formed by three rings embedded in the nuclear envelope (Fig 1): a nucleoplasmic, an inner, and a cytoplasmic ring. The NPC protein inventory, the nucleoporins, has been studied for diverse eukaryotes—e.g., trypanosomes, tetrahymena, fungi, plants, animals, and humans. Although sequence similarity is often low—unfortunately resulting in differently named nucleoporins—overall, structural conservation allows identification of 20 common nucleoporins, which are found in all eukaryotes, presumably forming the conserved core of protein interactions in NPCs [3]. This is probably best exemplified for the so-called Y-complex, a submodule of the NPC [4]. It is assembled from 6–10 nucleoporins, depending on the species, which are arranged in an eponymous Y-shaped structure first seen in the budding yeast *Saccharomyces cerevisiae* [5] and later confirmed in humans [6] and the fungus *Chaetomium thermophilum* [7].

**Fig 1:**
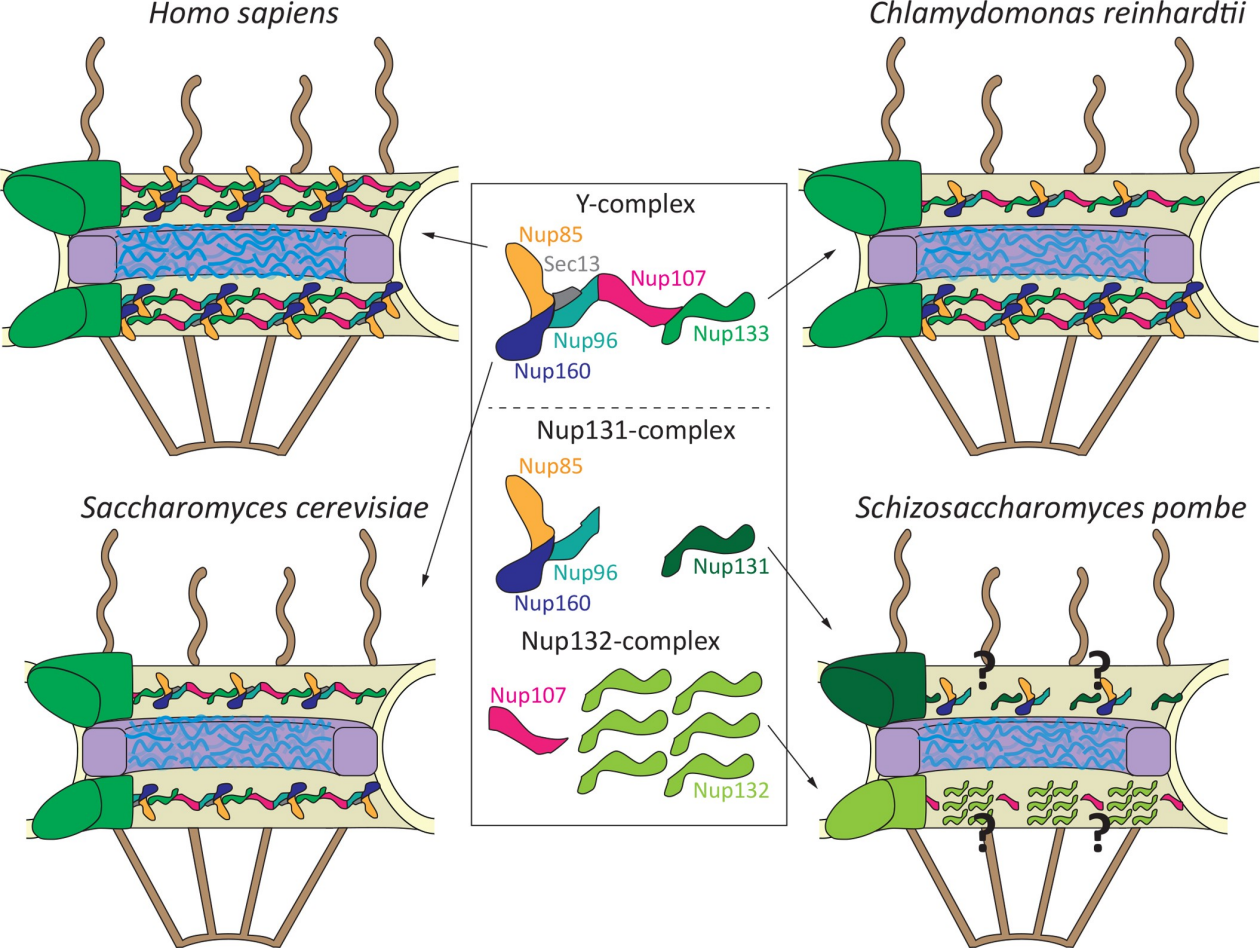
Y-complex arrangement in different NPCs. The basic NPC structure is arranged in three rings, the nucleoplasmic and cytoplasmic (green) rings and an inner ring (purple). The central part is occupied by a protein meshwork (blue) important for the transport and exclusion function of the pore. Cytoplasmic and nucleoplasmic extensions (brown) emanate from the respective ring structures. A prototypical Y-complex is shown with its core components, Nup85, Nup160, Sec13, Nup96, Nup107, and Nup133 (following the human nomenclature and lacking the nonconserved complex members). *Schizosaccharomyces pombe* Nup131 and Nup132 Y-complex derivatives are shown. For simplicity, the nonconserved components, including Nup37 and Ely5 in *S*. *pombe*, are omitted. Sec13, so far regarded as a conserved Y-complex component with a second function as a COP-II coat subunit, is present in *S*. *pombe* but does not localize to NPCs [13]. COP-II, coat protein complex II; NPC, nuclear pore complex; Nup, nucleoporin; Sec, secretory.

However, the view of an overall similar NPC structure has been recently challenged. The human cytoplasmic and nucleoplasmic rings are each formed by 16 copies of the above-mentioned Y-complex [6, 8], bringing the total number to 32 (Fig 1). In each of these rings, the Y-complexes form two concentric circles, and the same arrangement is found in NPCs of the clawed frog *Xenopus* [9]. In contrast, in budding yeast, the cytoplasmic and nucleoplasmic rings are each composed of a single eight-membered Y-complex circle, summing up to 16 Y-complexes per NPC [10], half of the number found in the human NPC. In the algae *Chlamydomonas reinhardtii*, the cytoplasmic ring contains eight Y-complex copies arranged in a single ring resembling the arrangement in budding yeast, whereas the nucleoplasmic ring consists of 16 copies forming two concentric circles, as in the human NPC [11].

Although Y-complex copy numbers in the rings and, thus, in the entire NPCs vary between species, the overall Y-complex structure seemed to be an evolutionarily stable arrangement [1, 3]. Yet a study from the Haraguchi lab in this issue of *PLOS Genetics* [12] indicates that the fission yeast *S*. *pombe* does not feel obliged to this least common denominator. Previous work [13] suggested that in this species, the nucleoporins forming the Y-complex do not show an equal stoichiometry, unlike in other organisms [10, 14]. A central component of the Y-complex, nucleoporin (Nup) 133, exists in fission yeast in two isoforms, Nup132 and Nup131 [15, 16], with Nup132 being about six times more abundant than Nup131 and all other Y-complex components [13]. How, then, do budding yeast NPCs incorporate Nup131 and Nup132?

Asakawa and colleagues [12] address this question by determining the relative position of enhanced green fluorescent protein (EGFP)/mCherry-tagged nucleoporins within NPCs expressed from their native promotors by high-resolution confocal light and immuno-electron microscopy. If one assumes that tagging does not influence localization because N- or C-terminal–tagged proteins show the same results, the surprising finding is that Nup132 and Nup107 (known as Nup84 in budding yeast) are located on the nucleoplasmic side of the NPC (Fig 1). In contrast, Nup131 and all other Y-complex components are found on the cytoplasmic side. Hence, the authors propose that in fission yeast, the cytoplasmic ring consists of eight copies of a reduced Y-complex lacking Nup107 and including Nup131 as a Nup133 homologue. The nucleoplasmic ring contains eight copies of Nup107 and 48 copies of Nup132 [12]. Consistent with an asymmetric distribution of Nup131 and Nup132 in the NPC, mass spectrometry identifies Nup131-interacting partners, which preferentially localize to the cytoplasmic side of the NPC. Indeed, in a test case, deletion of Nup131 results in loss of NPC localization of the candidate Far8, part of a nuclear envelope–localized protein phosphatase complex. Conversely, deletion of Nup132 causes a distorted NPC localization of Nup211, the homologue of human Tpr or budding yeast Mlp1p/Mlp2p, which are nucleoporins extending from the NPC structure into the nucleoplasm.

However, the same mass spectrometry analysis reveals that Nup131 and Nup132 can both interact with Nup107 and Nup85, which is in agreement with earlier work [15, 16] but in contrast to their proposed different positions and binding partners within NPCs. One might speculate that these interactions are only possible once the cells are broken up for the experiment. If so, what determines the correct and distinct localization of Nup131 and Nup132 and their respective exclusive interactions? As truncations, the C-terminal halves of both Nup131 and Nup132 localize predominantly to the nucleoplasmic side of the NPC, whereas the N-terminal halves as well as Nup131–Nup132 chimera combining the N- and C-terminal halves of the proteins largely mislocalize away from NPCs. This indicates that interactions with other nucleoporins might determine the precise localization within NPCs of Nup131 and Nup132. Forcing Nup107 to the cytoplasmic ring structure by fusion with Nup96 (Nup145C in budding yeast), a member of the fission yeast cytoplasmic NPC ring, targets a fraction of Nup132 to this NPC side. This argues that Nup107 is involved in localizing Nup132 in the natural situation to the nucleoplasmic NPC side but is not the only determinant.

In canonical Y-complexes, Nup133 is linked to Nup96 via the common binding partner Nup107 (Fig 1). As the cytoplasmic rings lack Nup107, it is unknown how Nup131 is integrated into the ring formed by the truncated Y-complexes. In the budding yeast NPC, the suggested ring-like arrangement of Y-complexes predicts an intersubunit interaction between Nup133 and Nup160 (Nup120 in fission yeast) [10, 17]. However, it remains to be seen whether fission yeast form a cytoplasmic ring structure by a head-to-tail arrangement of (truncated) Y-complexes. Alternative Y-complex arrangements have been suggested [18] and might be realized in the fission yeast NPC. Moreover, how eight copies of Nup107 and 48 copies of Nup132 arrange into a nucleoplasmic ring and whether the repetitive unit is indeed formed by six Nup132 copies and one Nup107 remain open. Finally, the Y-complexes are within NPCs’ major interaction hubs linking not only to nucleoporins of the inner ring but also to nucleoporins forming the cytoplasmic and nucleoplasmic NPC extensions. It will be interesting to learn whether these structures, especially the nucleoplasmic extensions, are affected by the unconventional Y-complex arrangement in fission yeast. Localization of several nucleoporins of the inner ring and cytoplasmic and nucleoplasmic extensions does not show deviations from the expected canonical pattern, but whether this also holds true for the protein interaction network remains to be seen. Ultimately, a cryo-electron microscopy structure of the fission yeast NPC will answer these questions, which is, in light of the novel *PLOS Genetics* study, a highly revealing and exciting research endeavor.

## References

[1] Beck M., and Hurt E. (2017). The nuclear pore complex: understanding its function through structural insight. Nat Rev Mol Cell Biol 18 (2), 73–89. 10.1038/nrm.2016.147 27999437

[2] Obado S.O., Field M.C., and Rout M.P. (2017). Comparative interactomics provides evidence for functional specialization of the nuclear pore complex. Nucleus 8 (4), 340–352. 10.1080/19491034.2017.1313936 28463551 PMC5597298

[3] Neumann N., Lundin D., and Poole A.M. (2010). Comparative genomic evidence for a complete nuclear pore complex in the last eukaryotic common ancestor. PLoS ONE 5 (10), e13241 10.1371/journal.pone.0013241 20949036 PMC2951903

[4] Kelley K., Knockenhauer K.E., Kabachinski G., and Schwartz T.U. (2015). Atomic structure of the Y complex of the nuclear pore. Nat Struct Mol Biol 22 (5), 425–431. 10.1038/nsmb.2998 25822992 PMC4424061

[5] Lutzmann M., Kunze R, Buerer A., Aebi U., and Hurt E. (2002). Modular self-assembly of a Y-shaped multiprotein complex from seven nucleoporins. EMBO J 21 (3), 387–397. 10.1093/emboj/21.3.387 11823431 PMC125826

[6] Bui K.H., von Appen A., Diguilio A.L., Ori A., Sparks L., Mackmull M.T., et al (2013). Integrated structural analysis of the human nuclear pore complex scaffold. Cell 155 (6), 1233–1243. 10.1016/j.cell.2013.10.055 24315095

[7] Thierbach K., von Appen A., Thoms M., Beck M., Flemming D., and Hurt E. (2013). Protein interfaces of the conserved Nup84 complex from Chaetomium thermophilum shown by crosslinking mass spectrometry and electron microscopy. Structure 21 (9), 1672–1682. 10.1016/j.str.2013.07.004 23954503

[8] von Appen A., Kosinski J., Sparks L., Ori A., DiGuilio A.L., Vollmer B., et al (2015). In situ structural analysis of the human nuclear pore complex. Nature 526 (7571), 140–143. 10.1038/nature15381 26416747 PMC4886846

[9] Eibauer M., Pellanda M., Turgay Y., Dubrovsky A., Wild A., and Medalia O. (2015). Structure and gating of the nuclear pore complex. Nat Comm 6, 7532. 10.1038/ncomms8532 PMC4491817 26112706

[10] Kim S.J., Fernandez-Martinez J., Nudelman I., Shi Y., Zhang W., Raveh B., et al (2018). Integrative structure and functional anatomy of a nuclear pore complex. Nature 555 (7697), 475–482. 10.1038/nature26003 29539637 PMC6022767

[11] Mosalaganti S., Kosinski J., Albert S., Schaffer M., Strenkert D., Salome P.A., et al (2018). In situ architecture of the algal nuclear pore complex. Nat Comm 9, 2361. 10.1038/s41467-018-04739-y PMC6006428 29915221

[12] Asakawa H, Kojidani T, Yang HJ, Ohtsuki C, Osakada H, Matsuda A, et al (2019). Asymmetrical localization of Nup107-160 subcomplex components within the nuclear pore complex in fission yeast. PLoS Genet 15(5), e1008061 Available from: 10.1371/journal.pgen.1008061. PMC6553703 31170156

[13] Asakawa H., Yang H.J., Yamamoto T.G., Ohtsuki C., Chikashige Y., Sakata-Sogawa K., et al (2014). Characterization of nuclear pore complex components in fission yeast Schizosaccharomyces pombe. Nucleus 5 (2), 149–162. 10.4161/nucl.28487 24637836 PMC4049921

[14] Ori A., Banterle N., Iskar M., Andres-Pons A., Escher C., Khanh Bui H., et al (2013). Cell type-specific nuclear pores: a case in point for context-dependent stoichiometry of molecular machines. Mol Syst Biol 9, 648 10.1038/msb.2013.4 23511206 PMC3619942

[15] Bai S.W., Rouquette J., Umeda M., Faigle W., Loew D., Sazer S., et al (2004). The fission yeast Nup107-120 complex functionally interacts with the small GTPase Ran/Spi1 and is required for mRNA export, nuclear pore distribution, and proper cell division. Mol Cell Biol 24 (14), 6379–6392. 10.1128/MCB.24.14.6379-6392.2004 15226438 PMC434257

[16] Belgareh N., Rabut G., Bai S.W., van Overbeek M., Beaudouin J., Daigle N., et al (2001). An evolutionarily conserved NPC subcomplex, which redistributes in part to kinetochores in mammalian cells. J Cell Biol 154 (6), 1147–1160. 10.1083/jcb.200101081 11564755 PMC2150808

[17] Seo H.S., Ma Y., Debler E.W., Wacker D., Kutik S., Blobel G., et al (2009). Structural and functional analysis of Nup120 suggests ring formation of the Nup84 complex. Proc Natl Acad Sci U S A 106 (34), 14281–14286. 10.1073/pnas.0907453106 19706512 PMC2732846

[18] Brohawn S.G., Leksa N.C., Spear E.D., Rajashankar K.R., and Schwartz T.U. (2008). Structural evidence for common ancestry of the nuclear pore complex and vesicle coats. Science 322 (5906), 1369–1373. 10.1126/science.1165886 18974315 PMC2680690

